# The practice of online assessment in an EFL context amidst COVID-19 pandemic: views from teachers

**DOI:** 10.1186/s40468-021-00143-4

**Published:** 2021-12-01

**Authors:** Nasim Ghanbari, Sima Nowroozi

**Affiliations:** Department of English Language and Literature, Faculty of Humanities, Persian Gulf University, Bushehr, 75169 Iran

**Keywords:** Online assessment, COVID-19, Barriers, English language teaching, Technology, Persian Gulf University

## Abstract

For many years, technology has been applied to improve the quality of language learning and teaching. However, the outbreak of COVID-19 pandemic accelerated the integration of technology in different language learning contexts. The sudden shift to online teaching faced educators with an array of challenges they had not experienced before. In particular, the teachers encountered many barriers with the online assessment of the students. To shed more light on what passed, following a qualitative mode of inquiry, the present study set out to find out how a group of 20 Iranian English language teachers at Persian Gulf University faced with the online assessment challenges posed by COVID-19. For this aim, the researchers conducted in-depth semi-structured retrospective interviews with the teachers at different times throughout the course. In addition, the teachers were asked to provide a narrative account of how they responded to the crisis. The analysis of the findings showed that after the shift to online assessment, the teachers initially faced with serious pedagogical, technical, administrative, and affective barriers, but as the course proceeded, they could adjust their practice with the new situation. However, the teachers recounted problems that still remained and negatively affected their practice. Overall, the study discusses that to improve the online assessment in the post-COVID-19 era several technological, pedagogical, and administrative measures should be taken into account. These would further improve the integration of the technology in the pedagogical context in the long run.

## Introduction

### Transition to online teaching

Within the past decades, numerous studies have shown the growing pattern of digital technology integration in different EFL/ESL learning contexts (Hafner & Miller, 2011; Mompean, 2010; Ros et al., 2010; Wu et al., 2010). However, despite the widespread use of technology in different language learning contexts, many scholars have counted several impediments for the effective application of technology in different language learning contexts (Burke, 2000; Egbert et al., 2002; Hedayati & Marandi, 2014; Jahanban-Isfahan et al., 2017; Marandi, 2010).

This state of technology integration in educational contexts coincided with the global outbreak of COVID-19 pandemic. Late in 2019, a new virus was observed in China. The virus which spread rapidly was labeled as COVID-19 by the World Health Organization (WHO) on February 11, 2020. Later, on 11 March, WHO declared the outbreak of a pandemic (Doctors without Borders, n.d.). Besides widespread disruptions to many sectors like travel, business, and economy, the pandemic has had unprecedented effects on education. This global transition from face-to-face education to fully online once happened at a time when there was no prior guidance, insight, or specific models of good practice for such a wide change. The sudden transition to remote teaching and learning using various internet-based resources created lots of challenges that students and teachers had to deal with (Turnbull et al., 2021). In addition, the idiosyncratic nature of the shift to online learning which considered online education not as a supplement to teaching but rather as a substitute for it have cast doubt on the relevance of the previous research on online learning.

The existing literature shows studies conducted on different aspects of the COVID-19 crisis. Drawing on the ABC-X model (Hill, 1949, 1958), Davies et al. (2020) provided a reflective account of the way experienced EAP practitioners in four Sino-foreign universities in China dealt with the crisis. The teachers were asked to reflect on five major themes of interaction, learner autonomy, feedback, leadership, and institutional support. The findings showed that the teachers should design online courses with support mechanisms to boost the learners’ autonomy. Also, increasing formative assessment and providing detailed feedback can enhance the quality of the students’ assignments. At the administrative level, the study recommends that program administrators avoid the one-size-fits-all approach to the new educational delivery mode and consider multiple factors in the success of the course. In another study, Hartshorn and McMurry (2020) investigated the effects of the COVID-19 pandemic on a group of ESL learners and TESOL practitioners in a university context in the USA. The findings of their study showed that both teachers and students experienced higher levels of stress after the pandemic to the extent that teaching and learning were not their main priority. Also, the students faced more challenges in the online mode. Khatoony and Nezhadmehr (2020) also investigated the challenges Iranian teachers faced in the integration of the technology for online classes during COVID-19 pandemic in Iran. The findings showed that the teachers, despite using the online applications and the platforms efficiently, faced many challenges such as lack of appropriate materials, learners’ lack of attention and demotivation toward online classes, lack of funding, and support for language institutions. However, the teachers were willing to the integration of the technology in the Iranian educational settings accelerated by the pandemic.

Todd (2020) also surveyed a group of 52 English language teachers at a known Thai university to explore their perceptions of the shift from the classroom to online teaching. The findings showed that while the teachers initially faced with many serious problems, they gradually found solutions to deal with them. Also, the teachers recounted some problems such as identifying suitable stimulating activities and assessing the students’ tasks that still remained.

In another study, Abid et al. (2021) studied the lived experiences of 11 Pakistani university teachers who participated in online teaching for the first time during the COVID-19 pandemic. The results of semi-structured interviews with the teachers revealed five themes of online teaching experience including culture and gender-related issues, teaching effectiveness, challenges in online teaching, coping strategies, and faculties’ post-COVID-19 perceptions. The findings emphasized that the faculty prioritized to focus on immediate online instructional matters in the wake of the pandemic with a lack of emphasis on global practices for online learning. However, the experience itself prepared the faculty for a blended learning approach and increased their awareness of global and future challenges.

In an attempt to identify the role of educational technologies in the transition from face-to-face to online teaching and learning activities during the COVID-19 pandemic, Turnbull et al. (2021) identified five challenges associated with online education experienced by higher education institutions: synchronous/asynchronous learning tool integration, access to technology, faculty and student online competence, academic dishonesty, and privacy and confidentiality. In addition, a thorough review of the literature in this study revealed several strategies for successful online implementation including providing e-learning training support for faculty and students, fostering online learning communities, and expanding traditional face-to-face course delivery to incorporate more elements of the blended learning.

### Online assessment

Assessment plays a key role in every pedagogical program. It monitors the quality of education and learning process. Moreover, by providing feedback on the students’ progress, it determines the extent the curriculum goals have been achieved. In fact, assessment activities are intended to measure whether the actual learning outcomes match the educational programs’ desired learning outcomes. In the words of Webber (2012, p. 202), assessment refers to “activities designed primarily to foster student learning.” Therefore, it is clear that the design and use of the assessment is an important part of the language teacher’s professional development. In fact, as teachers are frequently involved in formative and summative assessment of the learners in the pedagogical contexts, the way they operate and interact with larger contextual and experiential variables become important (Crusan et al., 2016; Zhang et al., 2021).

In accord with online learning practices, online assessment of the learners has been in place for many years. However, despite the application of online assessment in many pedagogical settings, many studies have reported barriers which affect the practice. In fact, the transition from the physical classroom-based traditional assessment to a virtual one caused by the outbreak of COVID-19 has had a far-reaching impact on different aspects of the classroom-based assessment. In this regard, many studies have investigated different variables such as lack of contact with the instructors, poor digital literacy, and lack of effective interaction and feedback mechanism (Holmes & Gardner, 2006; Kanaan et al., 2013; Masa’deh et al., 2013; Tarhini et al., 2013). Among different aspects involved in online assessment, understanding the way teachers implemented online assessment including their perceptions, the challenges they faced, and their coping strategies are important. The following are some of the studies conducted to clarify the role of the teachers amidst the COVID-19 pandemic online assessment.

Forrester (2020) studied the challenges and the possible solutions associated with moving a group speaking assessment task from face-to-face to online mode upon the outbreak of the pandemic at a university context in Hong Kong. The results showed that while teachers had a positive view on the new one-to-one discussion assessment, the students were ambivalent with some preferring the original group discussion mode. The study recommends that a body of administrative, pedagogical, and integrity concerns should be considered along with the teachers and the students’ feedback on the new assessment task. Abduh (2021) also investigated teachers’ perceptions of the assessment methods used in full-time e-learning during COVID-19 lockdown in a Saudi EFL context. The results showed that the teachers were ambivalent toward e-assessment. In addition, it was found that the teachers faced serious challenges in the online assessment of the students. In another study, Yulianto and Mujtahin (2021) examined the ELT teachers’ perspectives and their practices on the use of online assessment during COVID-19. Using open-ended questionnaires and online interviews, the authors found that the teachers were negative about the online assessment during the COVID-19 pandemic. In particular, they were concerned about the internet connection, the validity of their assessment, and the poor students’ enthusiasm. Despite these concerns, the study revealed that the online assessment helped the teachers in assessing the students’ achievement. Arif (2020) investigated the way English language teachers implemented online assessment and how they handled the challenges they faced when using online assessment during the COVID-19 pandemic. The results showed that the teachers employed different social networking tools such as Google Form and Google Classroom to assess the students’ online assignments. Moreover, the study showed that the teachers faced several challenges such as misunderstanding of the given instruction, internet connectivity, and difficulty in scoring. The study further showed that the teachers devised some strategies to cope with the problems they faced.

In the same vein, Chung and Choi (2021) explored how the transition to online mode affected the instructors’ teaching and assessment practices. They also studied the satisfaction level of the instructors and the students with the new online mode. The results of the study revealed that the teachers developed a professional learning community to develop new forms of assessment practices which were formative and process-oriented in nature. They also assigned multimodal projects to further promote sustainable assessment. The study further showed that while the students were highly satisfied with the new forms of language assessment practices, the teachers’ satisfaction level was low.

In an attempt to make assessment more authentic in both synchronous and asynchronous online assessment, Sutadji et al. (2021) utilized different forms of assessment such as a written test with case study questions/analysis, online discussion with peer assessment, and assessment of activeness by teachers. The findings of the study indicated a more authentic assessment was created as a result of conducting different assessment strategies.

Zhang et al. (2021) investigated the online assessment practices of six EFL teachers in a Chinese university. The findings of the study revealed that the EFL teachers made assessment decisions and selected specific assessment methods based on policy, the local context, and their own teaching experience and reflections.

## The current study

Long before the advent of COVID-19 pandemic, blended or mixed-mode delivery through the integration of the technology into the classroom teaching has been an option provided in many tertiary institutions. However, the provision of blended-learning was an additional provision and not as the whole replacement of the course. Therefore, since online mode of educational delivery has become the “new normal” in many tertiary education contexts around the world and also considering the power-coercive and unplanned nature of the change, a coherent research program is needed to investigate the change in different tertiary education contexts. In this way, the directions for the successful online courses in the future would be identified.

In addition, considering that the professional development of teachers happens in the situated contexts of practice, therefore, a greater understanding of the particular challenges the teachers faced in the online assessment would help policymakers in TESOL to improve the quality of online assessment programs in the local contexts of practice. Additionally, quality research on the lived experiences of the Iranian EFL teachers dealing with online assessment is lacking. Building on the above ground, the present study was conducted to answer the following two research questions in the particular context of the present study:

- *What were the barriers faced by Iranian EFL teachers involved in online assessment at the time of COVID-19 pandemic?*
- *What were the coping strategies of Iranian EFL teachers involved in online assessment at the time of COVID-19 pandemic?*

## Method

This section addresses the participants, the context in which the study was conducted, and the instruments used to collect data. It also explains the data collection and data analysis procedure used in this study.

### Participants

A body of 20 (11 female and 9 male) Iranian English language teachers participated in this study. The participants were selected following the convenience sampling procedure in which the researchers asked the available teachers who were qualified to participate in the study. Sixteen of the teachers held PhDs and four had MA degrees in English language teaching (Table 1). The teachers were eager to share their experiences of online teaching with the researchers. Therefore, they were asked to sign an informed consent to participate form. By so doing, the teachers agreed to contribute to the study. They were also assured that their data would be confidential and would be used only for research purposes. So, they actively participated in the whole study period.

**Table 1:** Demographics of the participants in the study

	Categories	***n***
**Gender**	Male	9
	Female	11
**Age**	38-45	14
	45-56	6
**Degree**	PhD	16
	MA	4
**Context of teaching**	PGU	20
**Teaching experience**	1-5 years	3
	6-12 years	5
	12-25 years	12

### Instruments

#### Interview package

A semi-structured interview protocol was prepared in order to solicit the views of the teachers. To develop the interview items, the researchers used the related literature and their experiences with online assessment. The interview items were prepared in Persian (i.e., native language of the participants) to prevent any possible language hindrances. The duration of the interviews differed depending on the topic discussed. Each interview session lasted about 2 h. On the whole, six interviews which totaled up to 750 min were conducted throughout the study. The interviews conducted and audio-recorded by the researcher were next transcribed for further analysis.

#### Teachers’ narrative accounts

The teachers participating in this study were asked to carefully note down how the rapid transition from the traditional face-to-face assessment to the online assessment mode affected different aspects of their usual evaluation practice such as their interaction with the students, feedback procedure, technical and practical support, and administrative concerns. These descriptive accounts were expected to provide details of the teachers’ practice who were involved in the online assessment. It was also hoped that the data provided in the accounts would further add depth to the findings of the interviews.

### Context of the study

The study was conducted in the department of English language and literature at Persian Gulf University in Bushehr located in southwest of Iran. In Iran, the second semester of the academic year runs from February to June. At the time of the study, the number of COVID-19 cases was relatively few in the province; however, the fear and anxiety caused by the pandemic was clearly observed in the campus and among the teachers and the students. Three weeks after the beginning of the second semester, social distancing rules came into effect across the country which meant that all face-to-face classroom teaching had to stop. However, the shift to online classes appeared with a delay. In fact, although the university had developed its learning management system (LMS) long before the pandemic, such a widespread and abrupt shift to online teaching was not expected. Also, despite many teachers had used some online tools to supplement their teaching, few of them had taught the whole course online. Needless to say, online assessment was quite new in the context. Following a brief suspension time which lasted for about 1 week, the university provided different departments including the English department with instructional videos and the needed technical support on how to use the LMS platform and the *moodle* for the new online teaching which had online assessment as an important part. Therefore, upon the university’s decision to stop all face-to-face instruction, English department also decided to continue the remaining 12 weeks completely online.

### Data collection

In order to collect data for the present study, the researchers developed a semi-structured interview which addressed salient aspects of the teachers’ assessment practices. Overall, out of the six focused interview sessions, three were done collectively with groups of the participants and the remaining three were conducted individually with each of the teachers. Critical points were recorded and discussed with the teachers in the interview sessions. The interviews were conducted at different times throughout the 12-week online sessions. Some of the interviews were conducted shortly after the shift to online sessions. Some others were conducted in the middle of the sessions which were after the Iranian New Year holidays and finally the teachers were interviewed after the final exams in June 2020. In addition to the interviews, the teachers were asked to write retrospective descriptive accounts of their online assessment experiences. Upon the end of the 12-week instruction, the teachers provided detailed accounts which showed their reflection on salient aspects of their practice.

### Data analysis

To analyze the data, qualitative content analysis procedure was used. The common practice in this mode of analysis is to analyze the data to the extent that categories, themes, and patterns emerge. Upon recording the interviews, they were transcribed verbatim and the researchers carefully read the transcripts several times. Finally, after multiple rounds of data analysis, the researchers could identify the main categories of challenges the two teachers faced in their assessment practices. In addition, the ways the teachers handled the challenging situations were identified and categorized. Moreover, in order to ensure the consistency of the coding, another experienced colleague was asked to code 20% of the transcripts. The inter-coder analysis revealed 80% consistency between the two coding.

The analysis of the teachers’ descriptive accounts was also conducted in the same way. For this aim, the researcher synthesized the accounts written at different times during the course. Upon several rounds of analyses, the main themes emerged from the accounts. The teachers were also asked to clarify the vague parts in their accounts to provide an accurate interpretation of them. Next, the themes were further analyzed to develop broader categories. The general categories that emerged from the reports were then used to triangulate the findings of the interviews. On the whole, the findings of the interviews and the teachers’ narrative accounts were used to identify the challenges the teachers faced and the solutions they came to throughout a 12-week of online teaching and assessment in the particular context of the present study.

## Results

### Investigating the first research question

Overall, the analysis of the six rounds of the interviews and also the teachers’ descriptive accounts revealed the following barriers the teachers faced in online assessment. In the following, each of these is explained.

#### Technological barriers

Under this theme, four sub-themes including digital literacy, technology access, attitudinal factors and academic dishonesty can be named. A rigorous analysis of the interviews and also the teachers’ reports revealed that early after the shift to online assessment, the teachers faced the greatest number of technological problems. Almost all the teachers stated that they were not familiar with the university learning management system (LMS). In fact, the teachers considered LMS which was developed by the university long before the pandemic as a supplement to their teaching rather than replacing all their teaching activities. Besides the technological struggles, the teachers could not trust in the technology as a reliable assessment tool. The following extracts describe the days after the shift to online assessment as so:

I did not know how to work with LMS, but worse I could not trust in it. I thought technology negatively affects the quality of classroom evaluation.I had to leave aside the repertoire of my assessment experiences obtained through long years of face-to-face classroom assessments.

Online assessment created several different concerns for me. For example, how to monitor students’ exam participation, how to deal with technological failures in a context with a long history of technological disruptions. I could not trust the online assessment of the students!

Gradually, as the course proceeded, the problems changed to be mostly technical and not attitudinal. One of the teachers, for example, stated that she did not know how to compress the video and audio exam files and then upload them in the site, so she missed many of the files she had worked on for hours.

In addition, another teacher described the final exam days when she was always worried about the blackout—which was common at that time of the year in the area—and the poor internet connectivity. She also counted times when during the exam session, some students were not able to connect to the microphone, or had problems with uploading their response files. Overall, technological barriers challenged the online assessment of the students in the context.

Academic dishonesty was the other major technological challenge with online assessment of the students. The teachers were worried about the security of the online exams and if the students were truly assessed. Moreover, they did not know how to develop tests to avoid the possibility of plagiarism to the extent possible. One of the teachers, for example, counted two challenges she faced when developing the final exams:

I could not type the test items in moodle directly. In addition, I had to change the multiple-choice items into the essay-type ones which took me a lot of time and effort!

In fact, the security of online exams or cheating issue was a major concern raised by many teachers. They were after finding solutions to lower the chance of cheating in the online exams. The teachers believed that the issue of academic dishonesty should be resolved at the university administrative level.

#### Pedagogical barriers

The sudden shift to online assessment created a number of pedagogical challenges for the teachers. In fact, many of the problems occurred at the interface of pedagogical concerns with the technical and administrative ones. For example, some of the teachers stated how they had to assess the students in the absence of physical interactions. Moreover, they referred to poor internet speed, lack of the students’ access to suitable devices (e.g., laptop or smart phones), poor feedback mechanisms for the during-the-course evaluations, and also, lack of higher-order institutional rules to integrate the online assessment practices in the university. Additionally, the teachers were worried about the washback effect of the online assessment on the students’ learning of the course content. The concern was that online assessment introduces new variables in the assessment context which can contaminate the assessment outcomes. In other words, the students’ performance in online tests were no more affected by only their knowledge of the particular construct, rather new test method facets moderate and affect their performance. In a context where online assessment was a new experience for both teachers and students, valid inference-making based on the students’ exam performance became difficult.

The other problem was the difficult task of developing and correcting the online exams. One of the teachers, for instance, mentioned:

Aside from long hours I spent for designing online exams, I had to sit in front of the monitor for many hours to correct the students’ online exams. The bright light of the monitor and long hours of sitting hurt my eyes and my back.

Adding to all these pedagogical problems was the lack of explicit codes of online testing issued by the university. The teachers did not know whether to adopt the previous face-to-face assessment mechanism which emphasized the summative assessment of the students or increase the load of formative assessment of the learners.

The other problem was that the particular characteristics of online assessment promoted a transmission education approach where in a teacher-fronted classroom the students only interacted with the teacher-examiner to receive the content and reflect on that. The poor interactive context of the online assessment undermined the transformative, participatory assessment practices such as peer-assessment practices.

#### Affective barriers

Teaching stress caused by the online assessment affected the teachers’ well-being and mental health. In fact, preparing online tests while considering the content, time, and situation of the students along with maintaining the work-life balance were the most affective challenges the teachers faced during the pandemic. Furthermore, the self-isolation that happened following the national quarantine order challenged the teachers’ mental health. As an evidence, one of the teachers who suffered from some background illness was particularly affected by the laborious online assessment activities. She stated:

My doctor was not available in the quarantine days. I was also under the pressure of preparing for online synchronous and asynchronous exams that took me much more time than the traditional exams. I really felt exhausted!

The teachers further stated that the self-isolation and the sad news they heard every day about the coronavirus made them scared and worried about the future. They confirmed that not socializing with others (i.e., students, colleagues, and friends) while being fully involved with the requirements of online assessment was difficult for them.

In addition, maintaining the work-life balance was a constant challenge especially for the female teachers. For example, some of the female participants stated that they had to manage their household chores which overlapped with their online classes. In addition, while some teachers considered the family as a supportive source that can alleviate stress level, others were worried about the health of the older family members as interactions between them might increase the number of COVID-19 cases.

#### Institutional barriers

What is expected is that at the time of crisis institutions provide clarity and stability to avoid anxiety and anarchy that might affect the normal performance. In addition to the domains that the university functioned well, the teachers referred to some institutional challenges that they faced. Regarding the unprecedented nature of the COVID-19 pandemic which caused a sudden shift to fully online courses, the expectation was that the university provided clear guidelines for online teaching including details of online assessment. As one of the teachers put it:

There was a two-week delay by the university to announce the details of the shift to online teaching policy.

Moreover, since there was no specific model to follow, the university announced guidelines about the synchronous and asynchronous online courses which were vague and they still added to the confusion. Some teachers, for example, questioned the weight of final exams. In a context where there was a serious concern for the security of the exams, the teachers expected a different assessment procedure in which formative assessment received more weight. The teachers also expressed that the university educational administration should provide further details about the final exams. One of the teachers, for example, pointed to some of these concerns:

Even in online mode, final exams should keep its place. There should a be a strict verification mechanism. There should be also alternative options at the time of technical failures. The university should make it clear how the students’ class participation throughout the course would be checked.

A related challenge to the above was the lack of online training (e.g., online workshops) for the teachers to professionally prepare them for the online teaching and assessment. The teachers in the study referred to the days they fixed the problems they faced in the class by trial and error.

Moreover, the teachers complained that while the university had posed strict control mechanisms on the teachers’ online teaching performance, the students were left alone and there were no rules to monitor their exam performance which in turn affected the validity of their test performance. One of the teachers, for example, stated:

I think both teachers and the students are involved in the assessment which means the pedagogical norms and disciplines should be observed by both sides. Unfortunately, the university did not monitor the students’ participation in online classes or their improper academic behavior. But the teachers’ performance is strictly checked every week.

The other administrative challenge the teachers faced was the lack of the university financial support to provide the needed equipment for the teachers. The teachers referred to the days when they had to experiment with different devices while each had technical problems. They confirmed that the university which was affected by the poor economic situation of the country could not provide financial support at this level; however, it was expected that some other options such as long-term loans be given to the teachers to provide their needed equipment. Apparently, the teachers working at self-isolation and dealing with many technical difficulties were disadvantaged by the unfair university rules.

In sum, the COVID-19 pandemic created some challenges at the institutional level for the teachers. The teachers in this study attributed these to the lack of a model at the time of crisis. However, they struggled to negotiate their ideas to the university leadership during the days of coronavirus.

### Investigating the second research question

As the course proceeded, the teachers could cope with the problems they had faced early after the shift to online assessment. In the following, some of the teachers’ initiatives will be explained. However, some challenging areas still remained until the end of the course.

#### Technological solutions

Some of the technological challenges that the teachers mentioned above proved not to be really problematic. The university provided proactive plans such as a number of instructional videos on different aspects of online assessment which were published for the university LMS site. Additionally, the university IT department actively helped the teachers and the students to resolve the technical problems they faced. The teachers also mentioned that they greatly benefited from the instructional videos provided by the IT department in their online exams. The teachers also gradually learnt to do all their evaluation in the online mode; hence, they tried to find solutions by either seeking support or experimenting different approaches which gradually changed their negative attitude to the online technology-based assessment that they had early after the shift.

Technological adaption was another coping strategy used by the teachers. Along with raising the load of formative assessment of the students, the teachers adopted both synchronous and asynchronous devices for assessing the students. The teachers also tried to improve their online competence by learning different technologies and finding the appropriate ones for their evaluation of the students.

Moreover, as the course proceeded, the teachers accepted that the technology has a downside. In fact, the reliance on devices, internet, electricity, and the other infrastructures were the requirements that might affect the quality of their assessment at any time. The teachers referred to these as the basic requirements of the online assessment.

#### Pedagogical solutions

After several weeks of teaching online, the teachers learned how to deal with the pedagogical problems associated with the online assessment. As an example, one of the teachers who did not receive any response from the students in asynchronous online classes decided to record her teaching and listen to them to find problems. He did it by several rounds of recording. Also, as the time passed the teachers could decide about the proper time for each exam type. One teacher, for example, explained how she devotes different times for different exams. She said as so,

This was not an easy task. Online exams deal with different constructs compared with the traditional exams. For example, online multiple-choice (MC) exams need more time than the traditional MC exams. This is because the online MC exams should be developed with higher difficulty levels but the time should not be as much that give the student the chance of cheating

The teachers also learned to receive feedback on their assessment using the online options. For example, the teachers used the polling option to receive the students’ feedback on the online exams. One of the teachers said so:

The students’ feedback gave me a sense of dynamism in the class. I could feel their presence and it reduced the weight of one-sided online assessment.

To enhance the security of the exams, the teachers used the LMS exam options of restricting the time of the exam and closing the exam page for a second review. In addition, the teachers could develop the exam based on a large repertoire of the exam type that the site provided for them. They also learned how to develop the essay-type items to decrease the probability of cheating in the online exams.

Although the teachers gradually adopted a healthy approach to online assessment and attempted to resolve the problems by experimenting and adjusting their teaching, some problems still remained. For example, the time allotted to correcting the online exams and the resulting boredom for the teachers along with the missing place of the institutional rules for organizing the online exams procedures were among the problems that the teachers reported facing until the end of the course.

#### Affective solutions

After the first few weeks of uncertainty and social isolation, the university took some measures which helped reduce the anxiety of the teachers. First, the institutional messages and plans of action communicated with the teachers gradually became clearer, detailed, and more consistent indicating the university administration desire to manage the crisis. This in turn reduced the stress level of the teachers considerably. The university offered free online counseling sessions for the students and the teachers. In fact, the university counseling center initiated a series of online workshops which addressed different areas of personal, familial, and job-related issues in the COVID-19 pandemic days. One teacher who participated in one of the online workshops about the family relations at the time of coronavirus explained how socializing with other colleagues who shared their experiences helped reduce her stress level. The other initiatives the university took were to start again the weekly meetings of the teachers in the online mode. Almost all the teachers in the study praised this act. They mentioned how hearing the other colleagues’ voices refreshed their depressed mood in those days of lockdowns.

Along with the institutional support, the teachers learned to exploit the potentials of social medias such as WhatsApp to expand the learning communities beyond the constraints of the synchronous online classes. In fact, social medias improved the teacher-students interconnectedness and in this way fostered a socially safe and engaging environment at the time of crisis.

#### Institutional support

After the nationwide policy of online teaching came into effect, the university administration began managing the situation. Despite the initial embarrassment which was reflected in the ambiguity of some announced instructional plans, the attempt was made to improve the quality of the online educational rules through communicating with different faculties. In fact, the university strengthened its connection with the teachers by asking them to consistently review the educational guidelines to further improve the quality of them. Moreover, as stated above, the university IT department initiated several online workshops to help the teachers how to carry out online teaching.

More specifically, the stated policy of the university on assessing students was to raise the ratio of formative assessment, encouraging the use of open-book exams, and also, the chance of make-up exams when the students missed the exams due to the practical constraints. In addition, the university was flexible as to which platform and what forms of the assessment the teachers used for the formative assessment of the students. Overall, the institutional policies were provided the sufficient freedom and flexibility for the teachers to have their individual assessment practices.

However, despite the above measures, there were still some rule vacancies in different aspects of online instruction. In addition, the university was unable to financially support the teachers with the devices they needed for the online instruction. These problems remained until the end of the course.

## Discussion

The results of the present study revealed that the pandemic influenced different aspects of assessment in the particular context of the present study. Exploring the way the teachers handled the different challenges they faced early after the shift to online assessment underscores the significant role of the teachers’ experiential knowledge in moderating the effects of any stressors that may threaten the functioning of educational systems (Zhang et al., 2021). Through adaptations and curriculum innovations, EFL teachers could overcome the obstacles they faced within the past decades (Jiang et al., 2019). This emphasizes the role of classrooms as fluid and dynamic contexts in which teachers have to adjust their assessment practices through enacting improvised changes.

The particular challenges the teachers faced and the solutions they came to upon experimenting with different aspects of the online assessment confirmed the dynamic role of the teachers in using the available sources to manage the teaching context in the new online mode. The findings of the study further showed that several problems that seemed difficult to the teachers at first, gradually disappeared and they could find solutions to them. This observation shows that the inhibiting attitudes of the teachers to online assessment gradually changed to the realistic position of taking advantage of the benefits of online assessment. Several factors account for the initial ambivalence of the teachers with regard to the online technologies. The first and the most important is the fact that the teachers were not technologically competent for the online assessment. In fact, although the university had developed its LMS long before the crisis, the teachers in this study just considered it as an option to supplement their evaluation of the students and not as a complete replacement of it. The second reason which is not unrelated to the first is the teachers’ uncertainty of the use of technology in their teaching in general. Even after several weeks of online instruction in this study, the teachers stated that face-to-face teaching was on the whole more efficient than the online teaching. In their ideas, the nature of technology removes a significant portion of the human interaction that must be replaced by hours of content preparation and assessing the students’ tasks. This can be confirmed by considering the problems that remained until the end of the course. In addition, the emotional burden caused by the sudden change in the medium of teaching considerably affected the teachers’ performance in early days of their online teaching. Following Makhwathana et al. (2017), a positive atmosphere provides a good platform for teaching and learning. Also, there is a direct relationship between teachers’ emotions and learners’ behavior and performance which means when the teachers express negative feelings, learners also feel humiliated, scared, and they might ultimately withdraw from learning.

However, a look at the coping strategies adopted by the teachers shows the gradual change which occurred in their attitude. In other words, it shows that despite the shift to online mode of work which happened at short notice, the teachers evaluated their resources and circumstances to cope with the new situation. For example, while the two teachers in this study seriously doubted the efficiency of online assessment, they gradually learned to use the benefits it provided to facilitate their assessment of the students. The above observations are in line with Zhang et al. (2021) who found that individual differences in teachers’ online assessment practices can be explained by experiential factors which included teachers’ motivation, attitudes toward new assessment methods, ability to handle online teaching and technology, and knowledge of assessment theory.

The challenges the teachers faced and the coping strategies they adopted in this study can be further discussed based on the ABC-X model (Hill, 1949, 1958). In fact, after the shift to online teaching, the situation was perceived as a crisis which threatened the teachers’ professional identity. However, gradually this perception of a full-scale crisis changed into a series of new challenges that should be managed. This new perception considerably reduced the stress level of the teachers who were scared, uncertain, and doubtful to the unknown future after the sudden shift to online mode. Although some of the challenges (e.g., pedagogical and administrative) remained until the end of the study, the teachers’ new attitude to the change helped them to cope with the new educational mode in an informed way. Moreover, the passage of time in this study showed that the teachers could gradually make a balance between what they were doing and their perceptions, actions, and emotions.

For many years, English language teachers have benefited from technological application in their classes (Hafner & Miller, 2011; Mompean, 2010; Ros et al., 2010; Wu et al., 2010). Many studies have also counted the benefits and obstacles of using technology in the ELT classrooms (Au-Yong-Oliveira et al., 2018; Bordbar, 2010; Hedayati & Marandi, 2014; Mahmoudzadeh, 2014; Marandi, 2010; Yadav et al., 2018). However, few studies have investigated the use of technology as a pedagogical resource at the time of crisis such as the COVID-19 pandemic. At a time when many educational institutions were forced to lockdown to avoid the spread of the disease, the integration of technology into evaluation system is very important. The way the teachers dealt with online assessment shows the degree they had managed the crisis. In this way, the findings of the present study are valuable as they show the efforts of a group of EFL teachers to handle the impediments of online assessment at the particular time of COVID-19 pandemic.

In sum, the findings of this study confirmed that the Iranian EFL context still suffers from numerous problems including the needed technological support, teachers and students’ online competence, teacher resources, and the learners’ constraints in using technology (Hedayati & Marandi, 2014; Marandi, 2010). Although the emergency teaching situation caused by the pandemic accelerated the integration of the technology in the local context, still there need concerted efforts for a smooth integration of technology in the EFL context of Iran. Provision of pre-service and in-service teacher professional development programs to improve and update the teachers’ knowledge and ability of using technology in their teaching along with providing the needed technological facilities in the context can considerably facilitate the state of online teaching in the context. Moreover, different learner-related variables such as digital literacy, degree of access to technological facilities, language proficiency level, age, gender, and more importantly their attitude to online teaching should be taken into account for removing the existing impediments in the integration of the technology in the context.

## Conclusions

The present study was conducted to provide insights into how a group of Iranian experienced EFL teachers caught in the midst of the COVID-19 crisis practiced the online assessment. The results of the interviews and the teachers’ descriptive accounts revealed that initially they were challenged on different technological, pedagogical, affective, and administrative grounds, but as the course proceeded, they found solutions to adapt their assessment practices in the new context. Although there remained some problems at the end of the course, the teachers continued their practice through dynamically experimenting and adjusting their assessment to the existing resources and circumstances.

The results of the present study have also a number of implications for the English language teachers. Since the educational system is at the beginning of exploring different aspects of the online assessment, the results of the present study help teachers to do their practice in an informed way and hence improve their practice in similar contexts in the future. Moreover, as the teachers faced challenges on different grounds, the university administration should provide constant training on how to use the technological devices in their assessment of the students and also train teachers on the effective use of technology in the design and development of the assessment tasks. The study further showed the agency of the teachers in implementing both the larger, national planned changes and also their classroom-based, improvised changes when implementing the online assessment. This finding emphasizes the role of teachers in the fluid and dynamic context of the classroom. The implication for policymakers is that teachers should be given autonomy because they can mediate among different variables at play in the context of classroom. The practice of online assessment in this study demonstrated that training programs should provide enough freedom for the teachers to implement their improvised changes as they are the only people who can address the realities of the immediate teaching context such as the students’ needs, classroom environment, and online resources along with the top-down educational policies. This requires a constant rapport between the teachers, students, and the administrators to continually negotiate the problems they face, so the suggested strategies would emerge from the actual context of practice.

Findings of the present study also provide new directions for future research projects. Different aspects of the challenges and the solutions emerged in this study can be separately studied in the large-scale research studies. As an example, teachers’ and students’ affective involvement in the online teaching can be studied to show the extent and quality of the emotion in the online teaching. As a next line of inquiry, the assessment practices of the teachers and the students can be also investigated to find out how the new test method facet of mode of delivery might affect the functioning of different stakeholders in the exam. Moreover, a large-scale survey study can be conducted to provide new insights on different aspects of online teaching.

This study also suffers from a number of limitations. First, the study was conducted in a single university in Iran. Future studies are recommended to include more institutions. In addition, the study was conducted with a limited sample of 20 teachers which restricts the generalizability of the findings to the particular context of this study. Also, to triangulate the results of this study, by using the findings of the present study, a survey can be designed and administered with a large number of the teachers. The results obtained in this way can determine how the teachers view the problems on a large scale. Moreover, in addition to the teachers, students can be examined for how they perceive the online assessment.

## Data Availability

The data associated with this study would be available upon request.

## Abbreviations

EAP: English for academic purposes
EFL: English as a foreign language
ESL: English as a second language
ELT: English language teaching
WHO: World Health Organization
PGU: Persian Gulf University
IT: Information technology
CALL: Computer-assisted language learning
LMS: Learning management system
TESOL: Teaching English to speakers of other languages
